# The Assessment of Animal Welfare in British Zoos by Government-Appointed Inspectors

**DOI:** 10.3390/ani2040507

**Published:** 2012-09-28

**Authors:** Chris Draper, Stephen Harris

**Affiliations:** 1 Born Free Foundation, 3 Grove House, Foundry Lane, Horsham, West Sussex, RH13 5PL, UK; 2 School of Biological Sciences, University of Bristol, Woodland Road, Bristol, BS8 1UG, UK; E-Mail: s.harris@bristol.ac.uk

**Keywords:** animal welfare, captive wild animals, government inspections, local authority, risk factors, Zoo Licensing Act

## Abstract

**Simple Summary:**

Since 1984, British zoos have been required to meet the animal welfare standards set out under the Zoo Licensing Act 1981. Zoos are regularly assessed by government-appointed inspectors, who report on animal welfare standards in each zoo. This is the first analysis of those reports from a representative sample of British zoos. We highlight a number of concerns about the inspection process itself, and identify areas where changes would lead to improvements in both the inspection process and our ability to monitor animal welfare standards in zoos.

**Abstract:**

We analysed the reports of government-appointed inspectors from 192 zoos between 2005–2008 to provide the first review of how animal welfare was assessed in British zoos since the enactment of the Zoo Licensing Act 1981. We examined the effects of whether or not a veterinarian was included in the inspection team, type of inspection, licence status of the zoo and membership of a zoo association on the inspectors’ assessments of animal welfare standards in five areas that approximate to the Five Freedoms. At least 11% of full licence inspections did not comply with the legal requirement for two inspectors. The inspectors’ reports were unclear as to how animal welfare was assessed, whether all animals or only a sub-sample had been inspected, and were based predominantly on welfare inputs rather than outcomes. Of 9,024 animal welfare assessments across the 192 zoos, 7,511 (83%) were graded as meeting the standards, 782 (9%) as substandard and the rest were not graded. Of the 192 zoos, 47 (24%) were assessed as meeting all the animal welfare standards. Membership of a zoo association was not associated with a higher overall assessment of animal welfare standards, and specialist collections such as Farm Parks and Other Bird collections performed least well. We recommend a number of changes to the inspection process that should lead to greater clarity in the assessment of animal welfare in British zoos.

## 1. Introduction

Captivity is widely acknowledged to affect the welfare of wild animals e.g., [1,2,3]. Zoological gardens, wildlife and farm parks and aquariums (hereafter zoos) may keep anything from a handful of wild animals from a few species, to tens of thousands of animals from hundreds of species [4]. Our unpublished analysis of zoo stocklists for a separate sample of 211 British zoos from 2003 to 2009 showed that they collectively held more than 60,000 individual wild tetrapods *i.e.*, mammals, birds, reptiles and amphibians: in this review we exclude domesticated species, fish and invertebrates. Given the variety of their species-specific requirements, monitoring the welfare of the wild animals held in British zoos presents a considerable challenge.

Zoos in Great Britain must be licensed by their relevant local governmental authorities (hereafter local authorities), who are responsible for implementing a system of inspections under the Zoo Licensing Act 1981 (hereafter ZLA), which came into force in 1984. Section 9 of the ZLA requires adequate standards for the accommodation, staffing and management for the proper care and welfare of the animals. This was subsequently amended to reflect the requirements of Council Directive 99/22/EC (“EC Zoos Directive”) [5], which requires that zoo animals are accommodated “*under conditions which aim to satisfy the biological and conservation requirements of the individual species, *inter alia*, by providing species specific enrichment of the enclosures; and maintaining a high standard of animal husbandry with a developed programme of preventive and curative veterinary care and nutrition*” (Article 3). Zoo regulation in Great Britain has been devolved to the three national governments, resulting in the Standards of Modern Zoo Practice [6] for England and the National Assembly for Wales Standards of Modern Zoo Practice (2004) for Wales. At the time of this study Scotland had not published its own national standards for zoos and was using those published by the Department for Environment Food and Rural Affairs (Defra) [6,7]. Since the national standards are very similar, hereafter they are referred to collectively as “Standards of Modern Zoo Practice” (SMZP).

The responsibility to monitor the welfare of the animals in a zoo lies with the staff, including keepers, curators and veterinarians, by undertaking informal daily animal assessments, welfare audits and regular reviews [8]. A sizeable minority of British zoos (93 at the end of 2009) are also members of the British and Irish Association of Zoos and Aquariums (BIAZA), a professional body “*representing the best zoos and aquariums in Britain and Ireland*” whose member zoos “*are leading the way in animal welfare; from promoting high standards in the daily management of animals, to supporting and undertaking research to increase our understanding of animal welfare and how to best promote it*” [9,10]. However, there is no requirement for prospective members to be inspected by BIAZA, and no formal inspection of members [11].

In addition to these internal welfare audits, British zoos are inspected by local authorities in conjunction with national government-appointed Zoo Inspectors (ZIs). Formal inspections include at least one ZI and occur at the end of the licensing period in order to renew the zoo's licence (renewal) and once during the licensing period (periodic). The zoo is given at least 28 days notice of an inspection, and told the names of the ZIs who will undertake the inspection; the zoo operator can appeal against any or all of the ZIs. The ZLA also includes provision for local authorities to carry out special inspections in any circumstances which, in their opinion, call for investigation. Further informal inspections are carried out by representatives of the local authority, generally Environmental Health Officer(s) or their equivalent. 

The three national governments in Great Britain maintain a two-part list of ZIs: Part 1 comprises veterinarians with experience of zoo animals, and Part 2 comprises individuals competent “*toinspect animals in zoos, to advise on keeping them and on their welfare … and to advise on the management of zoos generally*” [12]. The frequency of inspection and the number of ZIs required to participate in each inspection is determined by factors such as the size of the zoo and number of animals or species held (Sections 10 and 14, ZLA). While some zoos have a full licence, dispensations can be made under Section 14 of the ZLA for zoos with fewer individuals of conservation-sensitive species or a small range of species. However, all zoos should be inspected by at least one ZI on average every three years. Inspections are meant to last from most of one day up to three days [13]. Where an inspection is undertaken by more than one ZI, the inspection team may split up to cover different areas of a zoo [14]. While ZIs are required to take account of the SMZP when carrying out inspections [6], these include little information on how welfare should be assessed in zoos [15]. As a result, the SMZP are supplemented by the Zoos Forum Handbook, a non-statutory “living document” put together by the Zoos Forum, the government-appointed non-departmental public body advising on zoo licensing issues (it was replaced by the Zoos Expert Committee in February 2011). The Zoos Forum Handbook contains guidance and recommendations for zoos, ZIs and other stakeholders [6,15].

While ZIs are “*free to report on their work in any way they choose*” [14], 94% complete a non-mandatory questionnaire (form ZOO 2) to indicate their findings [16], copies of which are submitted to the local authority. Among other things, this form is intended to inform the local authority of the ZIs’ opinion regarding the conditions in the zoo, whether the zoo is meeting legislative requirements, and on recommendations to renew the zoo’s licence. There is also provision for the ZIs to recommend that the local authority attaches conditions to the zoo’s licence requiring the zoo to make compulsory improvements within a set time period to attain the required standards.

The first part of the process undertaken by ZIs is a pre-inspection audit of documentation relating to, among other aspects, the zoo’s animal records, programmes of animal husbandry and veterinary care and ethical issues. These assessments were not included in our analyses, although they may influence decisions made during the subsequent on-site inspection of the zoo.

For this analysis we focus on Sections 1 to 5 of form ZOO 2, which relate to the on-site inspection. These contain 48 questions that deal with the delivery of environmental parameters and animal management relevant to animal welfare corresponding to Sections 2.1 to 2.5 of the SMZP, which in turn approximate to the Five Freedoms [6,17] (see Table 1). The inspection form allows for YES, NO and N/A (not applicable) answers, with a comments box, to indicate whether the zoo meets the minimum standards for each question. There is also an optional grading system for YES answers, where 1 = Excellent to 4 = Barely Acceptable.

**Table 1:** Questions on form ZOO 2 relating to animal welfare that were assessed by ZIs during renewal and periodic inspections. The figures show the number of assessments in each category: n = 192 zoos. In Section 1, responses to question 1.6(a) do not tally with the responses to question 1.6: in some cases, question 1.6 was left blank but a response given to question 1.6(a), and in two cases, question 1.6 was marked NO but question 1.6(a) was marked YES.

	Yes (of which barely acceptable)	Yes but changes requested by addition of licence conditions	No	N/A	Left blank
*Section 1. Provision of food and water*					
1.1. Is each animal provided with a high standard of nutrition?	186 (0)	4	2	0	0
1.2. Is food and drink appropriate for the species/individual supplied?	184 (0)	4	3	0	1
1.3. Are supplies of food and water: (a) kept hygienically? (b) prepared hygienically? (c) supplied to the animal hygienically?	176 (3)	7	8	0	1
	160 (1)	19	11	0	2
	181 (1)	5	4	0	2
1.4. Has natural feeding behaviour been adequately considered to ensure that all animals have access to food and drink?	186 (0)	2	3	0	1
1.5. Are feeding methods safe for staff and animals?	188 (0)	0	4	0	0
1.6. Is feeding by visitors permitted?(a) If YES, is it properly controlled?	85 (0)	1	100	3	3
	77 (0)	2	9	66	38
*Section 2. Provision of suitable environment*					
2.1. Is each animal provided with an environment well adapted to meet the physical, psychological and social needs of the species to which it belongs?	160 (0)	14	16	0	2
2.2. Are the following environmental parameters appropriate: (a) temperature? (b) ventilation? (c) lighting? (d) noise levels? (e) any other environmental parameters?	179 (0)	5	7	1	0
	178 (0)	4	3	3	4
	179 (0)	3	7	1	2
	186 (0)	2	0	1	3
	163 (0)	8	4	9	8
2.3. Do animal enclosures have sufficient shelter?	179 (0)	5	7	0	1
2.4. Do animal enclosures provide sufficient space?	173 (2)	10	6	0	3
2.5. Are backup facilities for life support systems adequate?	132 (2)	5	11	43	1
2.6. Is the cleaning of the accommodation satisfactory?	181 (2)	5	5	0	1
2.7. Is the standard of maintenance adequate for: (a) the buildings? (b) the fences?	166 (1)	12	13	0	1
	157 (2)	7	17	8	3
2.8. Is all drainage effective and safe?	179 (1)	3	8	0	2
*Section 3. Provision of animal health care*					
3.1. Is each animal provided with a high standard of animal husbandry?	186 (2)	2	3	0	1
3.2. Do all animals on display to the public appear to be in good health?	186 (1)	0	6	0	0
3.3. Are observations of condition and health made and recorded?	171 (2)	2	17	1	1
3.4. Do all animals receive prompt and appropriate attention when problems are noted?	184 (0)	2	4	0	2
3.5. Are enclosures designed and operated in such a way that social interaction problems are avoided?	184 (0)	1	5	1	1
3.6. Are catch-up and restraint facilities adequate?	186 (2)	2	0	3	1
3.7. Is darting equipment satisfactory?	62 (0)	1	7	119	3
3.8. Are on-site veterinary facilities adequate?	135 (2)	3	29	23	2
3.9. Is each animal provided with a developed programme of preventative and curative veterinary care and nutrition?	150 (3)	8	32	1	1
3.10. Is a satisfactory programme of preventative and curative veterinary care established and maintained?	146 (2)	10	36	0	0
3.11. Is there a system for the regular review of clinical and pathological records?	135 (3)	6	48	0	3
3.12. Are appropriate veterinary records kept?	151 (1)	5	33	0	3
3.13. Are medicines correctly kept?	152 (1)	5	18	13	4
3.14. Are controlled drugs used and recorded satisfactorily?	56 (1)	1	6	129	0
3.15. Are appropriate antidotes available?	37 (0)	0	4	150	1
3.16. Are post mortem examination arrangements satisfactory?	148 (1)	12	29	3	0
3.17. Is adequate reserve accommodation available for isolation of animals for: (a) assessment? (b) treatment? (c) recovery? (d) quarantine (where required)?	162 (5)	8	17	3	2
	160 (3)	6	20	3	3
	161 (3)	7	18	3	3
	138 (3)	11	26	13	4
3.18. Are satisfactory measures in place to prevent the intrusion of pests and vermin into the zoo premises?	176 (2)	5	10	0	1
3.19. Does it appear that general sanitation and pest control are effective?	181 (4)	2	7	0	2
*Section 4. Provision of an opportunity to express most normal behaviour*					
4.1. Does accommodation appear adequately to meet the biological and behavioural needs of the animals?	171 (2)	11	9	0	1
*Section 5. Provision of protection from fear and distress*					
5.1. Are the animals handled only by or under the supervision of appropriately experienced staff?	186 (0)	1	4	1	0
5.2. Is physical contact between animals and the public consistent with the animals’ welfare?	173 (0)	2	3	10	4
5.3. Are interactions between the animals such that they are not excessively stressful?	184 (1)	3	1	1	3
*Section 10. Miscellaneous*					
10.4. Has an ethical review process been established?	116 (1)	11	63	0	2
*Section 12. Compliance check*					
12.3. Have existing licence conditions been met?	131 (1)	5	36	11	9

Two further questions on form ZOO 2 are of particular relevance to animal welfare: question 10.4 examines progress in establishing an ethical review process. Among other things, the ethical review process relates directly to animal welfare when focussing on “*situations where the use of animals (e.g., acquisition, management or disposal for conservation, education or research) may be in conflict with the best welfare interests of the animal or animals involved*” (Appendix 2.4, SMZP). Question 12.3 addresses compliance with conditions attached to the zoo’s licence following previous inspections, and so may serve as an indicator for the resolution of animal welfare problems.

While formal inspections by ZIs are just one part of a wider requirement for ongoing welfare assessment, and are relatively infrequent opportunities to assess overall welfare of the animals in a zoo, their strength is that they are independent evaluations, undertaken by individuals familiar with the legislation and the SMZP, and with knowledge of other zoos by way of comparison [15]. Consequently, they represent an important part of the process to monitor animal welfare within individual zoos and across the industry as a whole.

Our aims were to review the process and general results of the assessment of animal welfare standards in zoos in England, Scotland and Wales by government-appointed ZIs. In particular we: (i) review the delivery and process of zoo inspections; (ii) look at how many British zoos in our sample were judged to meet the minimum animal welfare standards 25 years after the ZLA came into effect; (iii) discuss whether particular types of zoo were associated with higher or lower animal welfare assessments; (iv) examine whether the inspection system is an effective method of assessing zoo animal welfare; (v) highlight areas where the inspection system should be improved; and (vi) discuss how this should lead to improved standards of animal welfare assessment in British zoos.

## 2. Experimental Section

Copies of the most recent inspection reports submitted for the zoos in their administrative area were requested from all local authorities in England, Scotland and Wales. We only included formal inspections, both renewal and periodic, undertaken between January 2005 and December 2008 under Sections 10 and 14(2) of the ZLA in the analyses; reports for informal or special inspections were excluded, as were any reports that did not use form ZOO 2. Informal inspections were excluded because they are often undertaken by local authority representatives, whereas the focus of the study was how government-appointed ZIs assessed animal welfare in British zoos. Special inspections may be undertaken for a variety of reasons, often in response to specific issues or concerns, and so their inclusion may bias the data. The few reports that did not use form ZOO 2 were excluded to ensure consistency of criteria being assessed.

We extracted information on: (i) the name of the zoo; (ii) the date of inspection; (iii) whether it was a renewal or periodic inspection; (iv) the type of zoo *i.e.*, Aquarium, Bird of Prey, Farm Park, General Mixed, Invertebrate, Other, Other Bird or Reptile/Amphibian [18,19,20], with zoos in Scotland classified following examples in England and Wales; (v) the type of licence (full or dispensation); (vi) the names of the ZIs who undertook the inspection and whether the ZIs included a qualified veterinarian (Part 1 of the ZI list); (vii) the outcomes reported by the ZIs to the questions in Sections 1 to 5 (those dealing directly with animal welfare), question 10.4 (the ethical review process) and question 12.3 (compliance with existing licence conditions), including the optional grading system where this was used; and (viii) whether a zoo was a member of BIAZA, or became a member during the year of inspection, based on BIAZA annual reports. Since the question whether the zoo permitted visitors to feed animals (Table 1, question 1.6) did not relate directly to animal welfare, we present the data but they are not included in the analyses, whereas control of feeding of animals by visitors (Table 1, question 1.6(a)) is pertinent to animal welfare and is included. 

We considered a zoo to be substandard for a particular criterion when the ZIs marked NO, or where the ZIs marked YES but then recommended that a condition for improvement to meet the standards for that criterion be attached to the licence. These YES BUT answers were included as substandard on the basis of the ZIs’ comments recommending conditions and/or details provided in the text of the condition. Cases where the ZIs marked YES but then suggested improvements in the comments box or at the end of the form were considered to meet the standards *i.e.*, comments and recommendations (as opposed to suggested licence conditions) were disregarded when categorising a criterion as substandard.

### 2.1. Statistical Analyses

The data were inputted into Microsoft Excel©. Multivariate analysis was considered unsuitable due to the limitations of the data, particularly the variability in the way ZIs appeared to interpret at least some of the questions, and so we only tried to identify general patterns. Since the data were not normally distributed, we used non-parametric statistics to identify factors associated with substandard animal welfare assessment in zoos. Data were analysed using SPSS® v. 16.0. The significance level for all tests was *P *< 0.05. Details of the tests used are given in the Results tables.

## 3. Results and Discussion

Copies of reports were received for formal (*i.e.*, renewal and periodic) inspections of 192 zoos (160 in England, 19 in Scotland, 13 in Wales) inspected between May 2005 and December 2008. 119 zoos were not members of BIAZA, 16 became members of BIAZA during the study period, and 57 were more longstanding members of BIAZA.

### 3.1. The Inspection Process

One inspection took place over two days; all other inspections were completed within a day. For those reports with both the names of the ZIs and the date of inspection (n = 173), one ZI was involved in the inspection of more than one zoo in a single day on 6 separate occasions *i.e.*, 12/173 inspections (7%) (Table 2). Section 10 of the ZLA requires that two ZIs carry out full licence inspections (*i.e.*, for zoos without dispensations). Of the 55 full licence inspections in the dataset, 43 (78%) were carried out by two ZIs as required; 6 (11%) were carried out by one ZI, and the local authority withheld this information for the other 6 (11%). The zoos where full licence inspections were carried out by one ZI were mostly General Mixed collections, keeping as many as 417 individual wild tetrapods from 77 species.

**Table 2:** Details of six occasions when two zoos were inspected by the same ZI on the same day.

Date	Distanceapart (km)	Type ofinspection	Type of zoo^1^	No. of animals^2^
12.06.2006	11.6	Periodic	Bird of Prey	161
		Periodic	Bird of Prey	n/a^3^
09.10.2006	18.3	Periodic	Bird of Prey	24
		Periodic	Other Bird	112
16.11.2006	4.8	Periodic	(Bird of Prey)	18
		Periodic	(Invertebrate)	106
21.03.2007	3.4	Periodic	Farm Park	115
		Renewal	Aquarium	5
06.07.2007	10.9	Renewal	Invertebrate	16
		Renewal	Other Bird	895
12.09.2008	36.0 (including a ferry crossing)	Renewal	(Bird of Prey)	n/a
		Renewal	(Aquarium)	n/a

^1^ Following Defra [18,19] and the Welsh Assembly Government [20]; brackets indicate zoos in Scotland that were classified following examples in England and Wales.

^2^ Excludes fish, invertebrates and domesticated species, since accurate stock figures were not available for these taxa, although they were included in the ZIs’ inspection.

^3^ n/a indicates that the number of animals held at the time of the inspection was not available.

Where full information on the ZIs, including names, was available (n = 176), a total of 59 combinations of ZIs (individuals or teams) undertook the inspections. Of 182 inspections where information was available, a Part 1 ZI was present for 148, with 34 inspections undertaken by Part 2 ZIs alone. Whether or not the zoo inspection team included a Part 1 ZI (*i.e.*, a veterinarian) did not affect the assessment of animal welfare standards in any of the five sections or overall (Section 1, U = 2,220, Z = −1.406, *P *= 0.16; Section 2, U = 2,182, Z = −1.334, *P *= 0.18; Section 3, U = 2,183, Z = −1.235, *P *= 0.22; Section 4, U = 2,384, Z = −0.900, *P *= 0.37; Section 5, U = 2,473, Z = −0.437, *P *= 0.66; overall, U = 2,469, Z = −0.171, *P *= 0.86).

### 3.2. Animal Welfare Assessment

Of the 9,024 questions across the 192 zoos, 7,511 (83%) were graded as meeting the standards, 782 (9%) as substandard and the rest were not graded (Table 1). The optional scoring system was employed for at least one criterion in 72 inspections (38%), and used to score 1,453/9,024 (16%) questions. Of these, 59 (4%) assessments were “barely adequate”, 316 (22%) “adequate”, 751 (52%) “good” and 327 (23%) “excellent”. “Barely adequate” assessments were scattered across welfare indicators (Table 1). Of the 192 zoos, 47 (24%) were assessed as meeting all the animal welfare standards in Sections 1 to 5; there was no significant difference in the proportion of BIAZA members and non-members that met all the welfare standards (G = 1.504, *P *= 0.22). Of the 47 criteria analysed, the number deemed substandard ranged from 0 to 21 per zoo *i.e.*, one zoo failed to meet 45% of the minimum welfare standards. For individual zoos, substandard assessment in one section was associated with substandard assessment in other sections (Table 3) *i.e.*, there was a dichotomy, with some zoos performing well, and others performing poorly, across a range of welfare criteria.

**Table 3:** Pearson correlations between the total number of criteria assessed substandard per zoo for each section of form ZOO 2: n = 192 zoos.

	Section 1	Section 2	Section 3	Section 4	Section 5
Section 1: Provision of food and water	-	0.434	0.448	0.327	0.406
		*P *< 0.01	*P *< 0.01	*P *< 0.01	*P *< 0.01
Section 2: Provision of suitable environment		-	0.467	0.505	0.262
			*P *< 0.01	*P *< 0.01	*P *< 0.01
Section 3: Provision of animal health care			-	0.331	0.245
				*P *< 0.01	*P *< 0.01
Section 4: Provision of an opportunity to express most normal behaviour				-	0.175
					*P *< 0.05
Section 5: Provision of protection from fear and distress					-

The maximum number of criteria deemed substandard in the 192 zoos was 6/8 in Section 1 (Provision of food and water), 6/13 in Section 2 (Provision of suitable environment), 12/22 in Section 3 (Provision of animal health care), 1/1 in Section 4 (Provision of an opportunity to express most normal behaviour), and 2/3 in Section 5 (Provision of protection from fear and distress). The type of inspection (renewal or periodic) had no significant effect on the assessment of welfare in any of the five sections or overall (Section 1, U = 3,455, Z = −0.483, *P *= 0.63; Section 2, U = 3,314, Z = −0.866, *P *= 0.39; Section 3, U = 3,400, Z = −0.542, *P *= 0.59; Section 4, U = 3,356, Z = −1.238, *P *= 0.22; Section 5, U = 3,437, Z = −1.136, *P *= 0.26; overall, U = 3,557, Z = −0.062, *P *= 0.95). ZIs assessed zoos with a full licence as having significantly better standards for the provision of animal health care (Section 3) than those with a dispensation under Section 14(2) of the ZLA (Table 4).

**Table 4:** Mean ± SD animal welfare performance by section for type of zoo licence. Mean animal welfare performance is the mean number of criteria assessed to be substandard per section *i.e.*, lower scores indicate higher levels of animal welfare assessment. The total includes all substandard criteria as an indication of overall performance. A G-test was used to compare Section 4, since there was only one criterion, Mann-Whitney tests to compare all other sections: n = 188 zoos.

	Full licence	Dispensation	Significance
	n = 55	n = 133	
Section 1: Provision of food and water	0.62 ± 1.22	0.38 ± 0.88	U = 3,389, Z = −1.043
			Ns
Section 2: Provision of suitable environment	1.16 ± 1.45	0.89 ± 1.39	U = 3,189, Z = −1.522
			Ns
Section 3: Provision of animal health care	1.49 ± 2.04	2.89 ± 3.17	U = 2,706, Z = −2.876
			*P *< 0.01
Section 4: Provision of an opportunity to express	0.11 ± 0.31	0.11 ± 0.31	G = 0.0047
most normal behaviour			Ns
Section 5: Provision of protection from fear and distress	0.05 ± 0.30	0.08 ± 0.37	U = 3,598, Z = −0.478
			Ns
Sections 1 to 5 combined	3.44 ± 3.95	4.35 ± 4.95	U = 3,321, Z = −1.002
			Ns

Of the 192 zoos, 25% were assessed as failing to meet all the standards for the provision of food and water (Section 1, Table 5), and 16% were judged substandard on the hygienic preparation of food (Table 1, question 1.3(b)). Feeding methods were found to be safe for staff and animals in 98% of zoos (Table 1, question 1.5). ZIs did not assess the standards in Section 1 (Table 6) significantly higher for BIAZA members than non-members. Feeding of animals by visitors was assessed to be inadequately controlled in 13% of the zoos where this was permitted (Table 1, question 1.6(a)). 

**Table 5:** Zoos that were assessed to meet the standards, or be substandard on one or more criteria in each section: n = 192 zoos.

	Met all standards	1 criterion substandard	>1 criterion substandard	No. of criteria assessed
Section 1: Provision of food and water	144	28	20	8
Section 2: Provision of suitable environment	105	37	50	13
Section 3: Provision of animal health care	67	33	92	22
Section 4: Provision of an opportunity to express most normal behaviour	172	20	-	1
Section 5: Provision of protection from fear and distress	183	4	5	3
Sections 1 to 5 combined	47	21	124	47

**Table 6:** Mean ± SD animal welfare performance by section for zoos that were and were not members of BIAZA. Mean animal welfare performance is the mean number of criteria assessed to be substandard per section *i.e.*, lower scores indicate higher levels of animal welfare assessment. The total includes all substandard criteria as an indication of overall performance. A G-test was used to compare Section 4, since there was only one criterion, Mann-Whitney tests to compare all other sections: n = 192 zoos.

	BIAZA members	Not BIAZA members	Significance
	n = 73	n = 119	
Section 1: Provision of food and water	0.29 ± 0.70	0.55 ± 1.13	U = 3,836, Z = −1.792
			Ns
Section 2: Provision of suitable environment	0.88 ± 1.33	1.03 ± 1.45	U = 4,098, Z = −0.724
			Ns
Section 3: Provision of animal health care	1.42 ± 1.97	3.10 ± 3.24	U = 2,983, Z = −3.737
			*P *< 0.001
Section 4: Provision of an opportunity to express most normal behaviour	0.08 ± 0.28	0.12 ± 0.32	G = 0.564
			Ns
Section 5: Provision of protection from fear and distress	0.00 ± 0.00	0.12 ± 0.44	U = 4,015, Z = −2.400
			*P *< 0.05
Sections 1 to 5 combined	2.67 ± 3.10	4.92 ± 5.23	U = 3,246, Z = −2.969
			*P *< 0.05

Of the zoos in the study, 55% met all the standards in the SMZP for the provision of a suitable environment (Section 2); 26% were assessed substandard on two or more criteria (Table 5). In this section there was no significant difference in assessments of BIAZA members and non-members (Table 6). 

In Section 3 (Provision of animal health care), ZIs reported that 35% of zoos met the SMZP for the provision of animal health care, with 48% judged substandard on two or more criteria (Table 5). BIAZA members performed significantly better than non-members (Table 6), whereas Farm Parks were judged to be worse than other types of zoo (Table 7). Animal husbandry, the health of animals on display, and catch-up and restraint facilities were reported as meeting the standards in 97% of zoos (Table 1, questions 3.1, 3.2 and 3.6). In this section, substandard assessments were made most frequently for the provision of adequate veterinary facilities (Table 1, question 3.8) and the provision and maintenance of preventative and curative veterinary care (Table 1, questions 3.9 and 3.10). Substandard performance was also commonly reported for record keeping and unsatisfactory arrangements for *post mortem* examinations (Table 1, questions 3.11, 3.12 and 3.16). The relatively high number of N/A responses to criteria in this section (e.g., Table 1, questions 3.7, 3.14 and 3.15) may have been due in part to inter-zoo differences in the species kept or facilities available. 

Section 4 (Provision of an opportunity to express most normal behaviour) had only one question: 90% of zoos met this standard. There was no difference between the assessments of BIAZA members and non-members in this section (Table 6).

ZIs reported a 95% compliance rate for Section 5 (Provision of protection from fear and distress) (Table 5). BIAZA members were assessed to perform significantly better than non-members (Table 6). Farm Parks, Other Bird and Reptile/Amphibian collections performed worst, although there were only two Reptile/Amphibian collections in the sample (Table 7).

**Table 7:** Effect of type of zoo on animal welfare performance in each section. The figures show the mean ranks for substandard assessments for each section by type of zoo, based on Defra's and the Welsh Assembly Government’s scheme for classifying types of animal collection [18,19,20]; zoos in Scotland were classified based on similar collections in England and Wales. Section 1: Provision of food and water; Section 2: Provision of suitable environment; Section 3: Provision of animal health care; Section 4: Provision of an opportunity to express most normal behaviour; Section 5: Provision of protection from fear and distress. Assessments of different types of zoo were compared with Kruskal-Wallis tests: n = 186 zoos.

		Mean rank					
Type of zoo	N	Section 1	Section 2	Section 3	Section 4	Section 5	Total
**Aquarium**	25	87.92	75.74	66.84	84.00	89.50	63.98
**Bird of Prey**	35	89.81	94.73	105.10	97.29	92.20	100.11
**Farm Park**	20	108.85	88.43	130.08	97.95	108.40	126.90
**General Mixed**	68	96.46	101.24	82.29	94.94	89.50	89.99
**Invertebrate**	9	70.50	75.11	100.11	94.33	89.50	84.89
**Other**	11	87.32	87.82	72.77	92.45	89.50	74.23
**Other Bird**	16	98.59	102.97	118.22	89.81	106.47	113.56
**Reptile/Amphibian**	2	70.50	120.00	125.50	84.00	89.50	116.50
**Significance**		X^2^ = 7.993	X^2^ = 7.834	X^2^ = 27.159	X^2^ = 4.654	X^2^ = 25.218	X^2^ = 20.680
		Ns	Ns	*P * < 0.001	Ns	*P < * 0.001	*P * < 0.005

### 3.3. Other Indicators of Animal Welfare

ZIs reported that 39% of zoos (excluding N/A and blank responses) had some problems with the establishment of an ethical review process (Table 1, question 10.4). However, the ZIs invariably gave a YES or NO answer, without any details on the nature of the problems. ZIs also reported that 24% of zoos were not complying with pre-existing conditions, which may relate to any aspect of their operation, not just animal welfare (Table 1, question 12.3). Assuming that these were attached to a zoo’s licence at the previous formal inspection, this would have been on average three years earlier. However, with the current inspection system, these conditions could have been attached at earlier inspections and so been in place for longer.

## 4. Conclusions

Our analysis complements earlier reviews by Greenwood *et al.* [16], which focussed on the performance of ZIs themselves rather than the ZIs’ assessments of the zoos, and ADAS [21], who reviewed the implementation of the ZLA in England and Wales by local authorities. Despite the recommendation that copies of inspection reports be sent to national authorities [6], only 59% of local authorities sent completed inspection reports to Defra and the Welsh Assembly Government [21], and there is no mechanism in place to gather and analyse data from these forms to examine zoo performance across the industry [22]. So this is the first analysis of the ZIs’ assessments of animal welfare standards in British zoos since the ZLA came into effect in 1984.

### 4.1. Data Quality

We analysed the ZIs’ assessments and were unable to comment on their accuracy. While no quantitative assessment of reliability has been published, they provide an overview of animal welfare standards in British zoos under the current legislative system because ZIs were familiar with the legislation and the SMZP and had the necessary expertise to compare between zoos [15]. No individual inspector will have had an undue influence on the analyses since the Defra list dated January 2008 included 19 Part 1 and 14 Part 2 ZIs, and the 176 inspections in this analysis where the names of the ZIs were available were undertaken by 59 different teams. Mechanisms to ensure inter-observer reliability currently consist of experience at recruitment, biennial training seminars, the provision of the Zoos Forum Handbook and initial “shadowing” of ZIs by new recruits [16]. Although we found no difference within sections between inspections including a Part 1 ZI and those only involving Part 2 ZIs, the comparison is limited because only zoos without a full licence could be inspected by Part 2 ZIs alone. However, only 4% of zoos questioned in 2003 expressed concerns regarding inconsistent knowledge between ZIs, and ZIs themselves did not identify animal welfare as a main area of the inspection process where inconsistencies arose [16]. 

Our analyses included inspections of a diverse group of zoos, covering all the types recognised by Defra [18,19] and the Welsh Assembly Government [20]. A complete list of licensed zoos in Britain was not available [7,19], but since Defra [19] listed 210 zoos in England, the largest of the three national authorities, our analysis included a substantial proportion of British zoos and so is likely to be representative of the industry. However, one of our key conclusions is that the system of zoo inspection and methods of reporting need to be improved. Under the current system it is impossible to determine whether substandard assessments applied to relatively few animals/enclosures in each zoo or indicated more widespread concerns, or conversely whether issues not judged to be substandard indicated good animal welfare provision. For instance, 26% of the graded assessments that met the minimum standards were only considered “acceptable” or “barely acceptable”.

Notwithstanding our comments regarding the actual inspection process, the data are representative of the situation that ZIs judged to exist under the prevailing regulatory system. While the data were not suitable for more detailed analyses, we believe they support the general conclusions presented here, and provide the first baseline measure against which to monitor future changes. This is an important step in improving animal welfare assessment in British zoos and thereby contributing to improving standards.

### 4.2. The Zoo Inspection Process

Despite assertions that zoo inspections generally range in duration from part of one day up to three full days [13], only one zoo inspection in this sample took place over more than a day, and 7% of the zoos were inspected on the same day by the same ZI. Although most of the 12 zoos involved were relatively small and close together, one had 895 wild tetrapods and some also held substantial numbers of invertebrates and/or fish, and possibly domestic animals. Since ZIs were required to include all these animals in their inspection, the stock figures in Table 2 are only a proportion of the animals that had to be inspected. In the previous round of inspections to those included in this analysis, two zoos in different cities 187 km apart, containing approximately 200 and 950 wild tetrapods, were inspected by the same ZI on the same day.

We only considered animal welfare issues in this review. However, ZIs are also required to examine conservation, public health and safety, and other factors, by visual inspection of the site and animals, and reviewing records and paperwork. Since ZIs are required to assess the individual welfare issues for a large number of animals from a wide range of species (the largest zoo in our sample had >2,800 individual wild tetrapods from 300 taxa), as well as conservation, public safety, legislative compliance and other issues, this would seem to place considerable demands on ZIs’ time, particularly when some zoos are inspected in less than a day. It also raises significant questions about the detail of formal animal welfare assessment for at least a proportion of British zoos. Furthermore, at least 11% of full licence inspections did not comply with the requirement for two ZIs, and 24% of zoos were reported as failing to comply with conditions imposed at the previous, if not an earlier, inspection. Similarly, 23% of local authorities reported experiences of zoos not complying with licence conditions and 10% reported delays to formal inspections [21]. Clearly the current licensing and inspection system does not prevent the continuation and/or recurrence of substandard conditions in British zoos. This compares unfavourably with other animal welfare inspections in Britain, such as under the Animals (Scientific Procedures) Act 1986, where inspectors grade infringements into four categories and have clear strategies in place for rectification and to prevent recurrence of problems [23]. In their review of the implementation of the ZLA by local authorities, ADAS also concluded that conditions attached to licences should be allocated an appropriate time span for implementation [21].

Questions to which ZIs report YES but then recommend that additional conditions are attached to the licence are problematic. In our analyses we combined these answers with outcomes marked NO because reporting YES but then stipulating conditions for improvement indicates that the situation did not meet the minimum standards. The ambiguity of these answers may indicate: (i) a tendency for ZIs to give zoos the benefit of the doubt; (ii) a reluctance to be seen to assess a zoo too harshly; (iii) a lack of independence of ZIs, a concern identified by Kirkwood [14] in his review of the operation of the ZLA; and/or (iv) the lack of clarity inherent in form ZOO 2, which does not make it clear whether responses refer to one, some or all the animals/facilities. Under the current system, if any aspect is substandard, e.g., the condition of one animal, even if the majority of the animal collection is in good condition, the ZIs should answer NO. Whatever the cause(s), employing an unofficial YES BUT outcome leads to a lack of consistency and clarity in the reporting process. 

### 4.3. Animal Welfare Assessment in Zoos

To achieve an effective and consistent assessment of animal welfare, the background knowledge of ZIs and the resources available to them should be as complete as possible. Greenwood *et al.* [16] found that ZIs “*may not be fully cognisant of the special needs of all species*”. This is not surprising, since there is a lack of biological and field data for many species held in zoos [24,25,26]. This makes it difficult to assess their basic welfare standards, let alone special needs. ZIs “*are encouraged to make full use of the latest Taxon Advisory Group or BIAZA (formerly called the Federation of Zoos) Guidelines and other sources when assessing exhibits. Zoos and experts in many parts of the world are developing guidelines and these should be referred to*” (SMZP, Appendix 8.4). However, at the time of this study BIAZA listed just 17 separate guidelines for species or taxa, covering nine genera of mammals [27]. Guidelines for other species or taxa had been published by the Association of Zoos and Aquariums and the European Association of Zoos and Aquaria, but only a minority of commonly-kept species had specific guidelines to which ZIs could refer. While the Association of Zoos and Aquariums was developing Animal Care Manuals for approximately 160 species or groups [28], only 5 had been completed [29]. To put this into perspective, there were approximately 384 species of mammal, 728 species of bird, 266 species of reptile and 85 species of amphibian in BIAZA-member zoos alone [30,31,32]. It is also unclear how these guidelines will contribute to improving the welfare of zoo animals. Guidelines or standards on animal management, husbandry or care tend to focus on resource inputs or “welfare potential”, even though welfare assessment must examine both inputs and animal-based outcomes [28]. It can also be unclear whether such guidelines are science-based, have been scientifically validated, or are based on expert opinion [26,28].

The Zoos Forum Handbook recommends that zoo assessments are based on a number of welfare indicators, including visual inspection of animals and records [15]. While it recognises that the role for direct welfare inspection by ZIs may be more suited to auditing operating systems, this is not reflected in form ZOO 2, nor in many of the responses reported. There is clearly an expectation that ZIs assess the welfare of individual animals. For example, question 3.2 asks “Do *all* (our italics) animals on display to the public appear to be in good health?”. The Zoos Forum Handbook outlines six behavioural indicators of welfare (approach/avoidance; stereotypies; overgrooming/self-harming; apathy; poor maternal care/infanticide; hyperaggression), seven physiological indicators of welfare (heart rate; cortisol; prolactin; neutrophil/lymphocyte ratio; reproductive hormones; body temperature change; weight change), and a number of parameters to use in a visual inspection of an animal’s physical state. Assessing the health of more than 2800 individual animals, *i.e.*, all the wild tetrapods in the largest zoo included in this analysis, is a lengthy process, even when relying just on visual measures. In general, no indication was given by ZIs as to which, if any, of these or other indicators were employed in reaching their assessment of the health of all the animals on display, nor whether this was done by direct inspection or by auditing the operating procedures.

While it is easier to assess provision, a range of standards for both provisions and outcomes are needed to assess welfare accurately; these include resources, management, records and welfare state [8,33,34]. Some recent studies have assessed animal welfare at the farm or group level e.g., [35,36,37]. However, there are substantial differences between on-farm or livestock systems, where large numbers of a single species with defined age (and often sex) cohorts are held in relatively standardised conditions, and zoos, which hold small groups of a wide range of species with different ages, ontogenies and specific husbandry requirements. Whilst assessing individual animal welfare must be the priority for zoo staff and veterinarians, individual welfare assessment is time-consuming and may not be suitable for a short duration, infrequent formal inspection system. It is more important that the formal inspection process incorporates a measure of the degree of compliance, rather than the current system of simple YES or NO answers to general questions encompassing all the animals in the zoo, which is basically group-level welfare assessment without any defined levels of acceptability, and as such is limited in its effectiveness.

It is also important to consider positive outcomes when assessing animal welfare. The EC Zoos Directive (Council Directive 99/22/EC) imposes a legal obligation to satisfy the biological requirements of animals in zoos, particularly to provide opportunities for them to express most normal behaviours. It is surprising, therefore, that unlike other sections of the reporting form, Section 4 only includes one question about provision of opportunities to express normal behaviour. While 90% of the zoos met the standard, this may simply reflect the lack of clarity. The section is entitled “Provision of *an opportunity* to express *most* normal behaviour” and the actual question posed is “Does accommodation *appear* adequately to meet the biological and behavioural needs of the animals?” (our italics). This subjectivity, and the focus on resource provision rather than welfare outcomes, suggests that this section in particular is inadequate to assess zoo animal welfare, and that ZIs should be required to include positive welfare outcomes in their assessments [38]. Similarly, while zoos performed relatively well in Section 5 (Provision of protection from fear and distress), the criteria assessed are arguably limited. Given the increasing body of evidence on the impact of visitor presence on the welfare of zoo animals (see [39] for review), factors other than just physical contact between visitors and animals (question 5.2) should be assessed.

### 4.4. Differences in Animal Welfare Assessments Between Zoos

A quarter of a century after the ZLA came into effect, it might reasonably be expected that there would be a high level of compliance with the minimum animal welfare standards in British zoos. Hence we focussed our analyses on the proportion of zoos that ZIs considered did not meet these minimum standards. Where a zoo was graded as not meeting these minimum standards, the current inspection system did not enable us to determine the proportion of animals involved. Even though these are minimum standards and many are vague, only 24% of the zoos were assessed to meet all the animal welfare standards, ≥95% of zoos met the standards for only 16 of the 47 animal welfare criteria, and one zoo failed to meet 45% of the animal welfare standards. Furthermore, substandard assessments in one section of form ZOO 2 were associated with substandard assessments in other sections, showing that zoos that performed badly did so across a range of animal welfare measures. 

While BIAZA members were expected to perform significantly better than non-members on most broad measures of animal welfare, this was not the case in three of the five sections, particularly Section 2 (criteria relating to the Provision of a suitable environment), where BIAZA members were as likely to be judged substandard as non-members. BIAZA members only performed significantly better than non-members in Section 3 (The provision of animal health care), the area where zoos generally were assessed to be worse, and Section 5 (Provision of protection from fear and distress). So membership of BIAZA did not indicate overall higher standards of animal welfare. 

The reasons for the differences in assessment between types of zoo are unclear. Farm Parks with zoo licences may have performed particularly badly because they originally focussed on domestic species and so their expertise and/or resources were insufficient to ensure that welfare standards were met for more exotic species. Other specialist collections (Other Bird, Reptile/Amphibian) also performed poorly. Although it was beyond the scope of this study, the range of species and/or number of individual animals kept by zoos deserves further investigation as a risk factor for animal welfare performance.

### 4.5. Animal Welfare Implications

The SMZP and current zoo licensing system can only play a part in the assessment of animal welfare in British zoos, much of which must be undertaken by the zoos themselves and by industry membership bodies such as BIAZA. However, the lack of clarity as to how animal welfare in British zoos is assessed by ZIs, coupled with the levels of non-compliance with both the inspection process and conditions imposed on zoos, raises significant concerns about the delivery of the zoo licensing and inspection systems. Under the current system it is impossible to determine whether substandard assessments applied to relatively few animals/enclosures in each zoo or indicated more widespread concerns, or conversely whether issues not judged to be substandard indicated good animal welfare provision.

Our analyses indicate that the following changes to the inspection process should lead to substantial improvements in the assessment of zoo animal welfare:

1. Auditing zoo records for accuracy and consistency is important, but should be a separate part of the inspection process. Simply summarising a zoo’s own welfare assessments during the inspection process adds little to the overall assessment of zoo animal welfare. An inspection should be an independent review in which ZIs are required to record which indicators were used to assess animal welfare.
2. At the moment it is impossible to gauge the criteria used in zoo animal welfare assessments, or the proportion of animals in the collection that were assessed. Form ZOO 2 should include details of the indicators used to assess animal welfare. If all the animals in the collection and their records were not inspected, form ZOO 2 should require ZIs to include details of the level of sampling employed, and whether the sample was random or focussed on particular taxa.
3. Additional guidance should be provided to ZIs on the suitability of indicating YES to a particular criterion but then recommending an additional condition for improvement be attached to the zoo’s licence, and on the suitability of indicating NO when this only applies to a part of the zoo or one or a few animals.
4. ZIs used the optional grading system erratically and infrequently. Making it mandatory to use the current or some other grading system to indicate the level of variability in welfare assessments, and form ZOO 2, will provide a baseline against which changes can be monitored over time. It should also be clarified when “Acceptable” and “Barely acceptable” should be used.
5. Section 4 of form ZOO 2 in particular needs to be expanded to allow a better assessment of whether animals can express normal behaviour, and inspections by ZIs should assess welfare outcomes as well as provision of resources. Similarly, Section 5 of form ZOO 2 needs to be expanded to include additional criteria that take account of the impact of visitor presence on the welfare of zoo animals, as well as physical contact between visitors and animals.
6. There is an urgent need for the development of, and validation of, science-based species-specific guidelines for the care of animals in zoos. Allied to this, a review of the criteria being assessed would enable advances in veterinary and other standards to be reflected in the ZIs’ reports.
7. The current inspection system appears to be superficial; it is rare for an inspection to last more than one day irrespective of the size and/or complexity of the zoo, and 7% of zoos were inspected by the same ZI on the same day. Clearer rules are required as to the time that should be spent inspecting a zoo, based on the size and type of collection, to ensure sufficient scrutiny of records, facilities and individual animals.
8. We analysed 47 questions that related to the assessment of animal welfare in British zoos. Of necessity, we had to treat all questions as being of equal importance. Weighting the assessment criteria would complement an improved system of grading infringements. Such a weighting system should be developed using a panel of experts and consensus techniques to ensure objectivity and would complement an improved system of grading infringements (see point 10).
9. The type of zoo as classified by Defra [18,19] and the Welsh Assembly Government [20] provides a useful indicator of zoos more likely to have substandard animal welfare assessments, and inspection frequency, detail and follow-up should be increased in zoos such as Farm Parks, Other Bird and possibly Reptile/Amphibian collections. Similarly, past welfare assessments could be used to identify zoos where more detailed assessments should be undertaken. There should also be different reporting forms for different types of zoo, particularly Farm Parks, Aquariums and other specialist collections, a recommendation also made by ADAS [21].
10. Since 24% of the zoos in our sample were not complying with conditions imposed some time earlier, a better system of reporting and enforcing conditions needs to be in place. The Animals (Scientific Procedures) Act 1986 provides a useful example of how infringements could be graded and addressed [23].
11. There should be a regular national analysis e.g., quinquennial, of all zoo inspection reports to monitor the working of the ZLA, highlight strengths and weaknesses of the inspection process, monitor the welfare assessments for zoo animals, and eliminate the apparently high levels of non-compliance with various aspects of the inspection process. For this it should be mandatory for ZIs to send their reports to the national authority or, more appropriately, the reporting system should be computerised to facilitate data analysis.
12. Removal of the zoo operator's right to appeal against any or all of the ZIs chosen to undertake an inspection, and independent auditing of the zoo inspection process and/or accreditation of the inspectorate in line with internationally-recognised standards would provide additional assurances of competence, consistency and impartiality.
